# Efficacy and safety of thrice weekly DOTS in tuberculosis patients with and without HIV co-infection: an observational study

**DOI:** 10.1186/1471-2334-13-468

**Published:** 2013-10-07

**Authors:** Richa Vashishtha, Krishna Mohan, Bhagteshwar Singh, Satish K Devarapu, Vishnubhatla Sreenivas, Sanjay Ranjan, Deepak Gupta, Sanjeev Sinha, Surendra K Sharma

**Affiliations:** 1 Department of Medicine, All India Institute of Medical Sciences, Ansari Nagar, New Delhi 110029, India; 2 Department of Biostatistics, All India Institute of Medical Sciences, Ansari Nagar, New Delhi 110029, India

**Keywords:** Antitubercular agents/therapeutic use, Tuberculosis/drug therapy, Mortality, HIV infections/complications, Treatment outcome

## Abstract

**Background:**

Despite the latest World Health Organization guidelines advocating daily therapy in HIV-TB co-infected individuals, there are few recent studies comparing outcomes of thrice-weekly anti-tuberculosis treatment in HIV-positive and HIV-negative patients with TB. The present study sets out to compare TB treatment outcomes in these two groups in the Indian national programme, which currently involves thrice-weekly therapy for all, regardless of HIV status.

**Methods:**

HIV-positive and HIV-negative were consecutively screened for enrolment into this prospective observational study, carried out at the All India Institute of Medical Sciences hospital, New Delhi, India, between 2006 and 2010. Patients were given short-course thrice-weekly rifampicin-based therapy, with all HIV-positive patients being started on highly active antiretroviral therapy at least 14 days after commencing TB treatment. Patients were regularly followed-up for 24 months after completion of treatment.

**Results:**

150 HIV-positive, 155 HIV-negative patients were enrolled consecutively for the study. Significantly higher treatment success (93.5% vs. 76.7% at end of treatment, p < 0.001) and lower mortality (2.8% vs. 21.6% on follow up, p < 0.001) were observed in HIV-negative patients. No significant difference was found in treatment failure (p = 0.16), sputum smear (p = 0.58) and culture conversion (p = 0.55), and non-serious adverse event incidence (p = 0.851) between the two groups. Low baseline CD4 cell count (<100 cells/ mm^3^) was the only predictor of mortality in HIV-TB patients (odds ratio 8 · 43, p = 0 · 013).

**Conclusions:**

Thrice-weekly anti-tuberculosis therapy is more effective in HIV-negative than in HIV-positive patients. However, outcomes in this HIV co-infected cohort were found to be similar to those reported previously with daily therapy, with no safety concerns. This should prompt further study into whether intermittent or daily therapy should be used universally in resource-poor settings, using large well executed randomised controlled trials.

**Trial registration:**

NCT No. 00698334

## Background

Tuberculosis (TB) and human immunodeficiency virus (HIV) infection are leading causes of morbidity and mortality worldwide [1,2]. Despite significant progress in TB control in recent years [3], the HIV epidemic has posed a substantial challenge in the form of HIV and TB co-infection [3-5]. There is much debate over how anti-tuberculosis therapy (ATT) should be administered to such individuals, given their immunocompromised state, potential for drug-drug interactions [6,7], higher mortality rates [1-4,8-10] and differences in presentation and organ involvement [3,6,7]. Some areas are widely agreed upon, such as modification of antiretroviral therapy (ART) to avoid interactions [3,4,6,7], ATT commencing before ART in treatment-naïve patients [3,6], and avoidance of once- or twice-weekly dosing schedules [2,3,6,11]. However, specific issues which are being increasingly debated and researched include optimal duration of therapy [3,6-8,11-15], use of daily versus intermittent regimens (WHO recommend daily in at least the intensive phase) [3,15,16], and optimal timing of ART commencement [2,3,5-7].

A recent meta-analysis by Khan and colleagues suggests that there is a relative lack of high quality data on outcomes of ATT in HIV-TB co-infected patients, especially with regards to intermittent dosing schedules [16]. When comparing daily and thrice-weekly therapy, the comparative meta-analysis included over 3000 patients who had received daily treatment in the intensive phase, but only 211 who were treated with thrice-weekly regimens [16]. There are many studies reporting outcomes of ATT in HIV-infected patients with sputum smear and culture positive pulmonary TB [2,10,13-15,17-19] but few including smear-negative and extrapulmonary TB [8,9,11,12]. In the real-world, co-infected individuals are much more likely to have these challenging forms of TB [1,3], thus highlighting lacunae in research.

It is our personal observation that TB treatment using the current Indian national guidelines, which, in line with the previous WHO guidance, advocate 6-month thrice-weekly ATT irrespective of HIV status, outcomes are poorer in HIV-infected individuals. Hence, an observational study was undertaken to compare outcomes of current TB treatment in ATT- and ART-naïve HIV-positive and HIV-negative patients.

## Methods

### Study design and case selection

This prospective observational study was conducted at the All India Institute of Medical Sciences hospital, a large tertiary centre in New Delhi, northern India. The Department of Medicine provides specialist TB and HIV services to Delhi and neighbouring states. All socioeconomic strata are represented, but the majority are in lower groups [20].

ATT and ART naïve patients aged 18–65 years receiving a new diagnosis of active TB between April 2006 and October 2010, were consecutively assessed for enrolment. The following exclusion criteria were applied: patients with previous history of TB, ATT or ART; pregnant, lactating and seriously unwell patients; those with significantly raised serum transaminase levels or concomitant renal impairment, liver failure, diabetes mellitus, epilepsy or alcoholism; patients with hepatitis B or C virus co-infection; those unable to attend follow-up; and any patient having been on ATT for more than two weeks during the current schedule.

Two groups were formed: one with ART-naive patients having a new or existing diagnosis of concurrent HIV infection, and HIV-negative patients in the other. The All India Institute of Medical Sciences Institute Ethics Committee approved the study, and written informed consent was obtained from patients.

### Diagnosis and baseline assessment

All patients were counselled and tested for HIV using a licensed third generation ELISA kit as described previously [20]. TB was diagnosed using investigations as indicated and possible. These included Ziehl Neelson smear microscopy followed by Lowenstein Jensen culture of sputum and any body fluids, secretions, tissue or pus; *Mycobacterium tuberculosis* polymerase chain reaction (PCR); histo- and cytopathological examination of tissue or fluid (including broncho-alveolar lavage if required); and various imaging modalities. For example, all subjects with suspected pulmonary TB underwent sputum or broncho-alveolar lavage smear and culture, whereas if fluids such as cerebrospinal fluid and pleural aspirate were smear negative, then *M. tuberculosis* PCR was performed.

TB diagnosis in suspected cases was classified as definitive (diagnosed by smear, culture or PCR) or probable (clinical and radiological response to treatment and suggestive radiology, histology, cytology or fluid biochemistry) [21].

All patients underwent baseline clinical assessment, chest radiography and blood tests, including CD4 lymphocyte count by flow cytometry (Becton Dickinson, USA). Plasma viral load estimation was performed in HIV-infected individuals, using either semi-quantitative (Amplicor HIV-1 Monitor Test, Roche, Indianapolis) or real time PCR methods. In pulmonary TB, chest radiographic severity was graded [22]. In smear-positive patients, the sputum smear bacillary load was graded as scanty, 1+, 2+ or 3+ as per WHO guidelines [23].

### Treatment

Thrice-weekly rifampicin-based directly observed ATT was given as per the Indian Revised National TB Control Programme guidelines [24]. This involved two months of thrice weekly isoniazid (600 mg), rifampicin (450 mg; 600 mg if weight > 60 kg), pyrazinamide (1500 mg) and ethambutol (1200 mg) [intensive phase], and four months of thrice weekly isoniazid and rifampicin (doses as above) [continuation phase] [24].

Extension of ATT was considered in patients who showed clinical and/or radiological signs of disease progression or partial response, or persisting sputum smear positivity at the end of the intensive phase, after a consensus decision by two or more investigators. The extensions were of one, two or three months, of either the intensive (one month) or continuation phase (maximum three months). If despite extension, a patient was still deemed to have incomplete response at the end of treatment, this was labelled as treatment failure. They were started on 'category II' ATT as per the national guidelines (i.e. longer therapy with streptomycin added) [24], subsequently guided by susceptibilities from susceptibility testing results.

All HIV-positive patients received free highly active ART as per India’s National AIDS Control Organisation guidelines: lamivudine (150 mg q12h) and efavirenz (600 mg qd) with either zidovudine (300 mg q12h, in those with haemoglobin >8 g/dl) or stavudine (30 mg q12h, if Hb <8 g/dl), which was started two weeks or more after commencement of, but during, ATT [25]. After completion of ATT, efavirenz was replaced with nevirapine (200 mg q12h) [25]. All HIV-infected patients diagnosed in 2008 or later were started on prophylactic co-trimoxazole therapy as per changes in national guidelines [25]. Of those diagnosed before 2008, all with CD4 count <200 cells/mm^3^ received prophylaxis.

### Follow-up

Follow-up was fortnightly during the first two months of treatment, subsequently monthly up to the end of ATT, then at 3, 6, 12, 18 and 24 months post-ATT. Follow-up, using a structured pro forma document, included clinical assessment, monitoring for opportunistic infections (in HIV-positive patients), routine blood investigations, sputum smear and culture if appropriate and possible, and repeat imaging as at enrolment to monitor response to treatment.

In pulmonary TB patients, monthly chest radiographs and fortnightly sputum smear and culture were performed.

CD4 cell counts were measured at enrolment and 6 months after starting treatment in all, with subsequent six-monthly counts in HIV-positive patients only. Plasma viral loads were measured at baseline, 6 months after commencing ATT, and then after a further 6 and 24 months in the HIV-positive group.

Adherence to treatment was checked at each visit to the DOTS (thrice weekly in the intensive phase, weekly thereafter) or ART centre (monthly) by questioning and blister pack checks. These were supported by a medical social worker.

Adverse events observed were noted and communicated to the principal investigator and ethics committee. These were classified as non-serious and serious adverse events. The former included mild and moderate events such as nausea, abdominal pain and itch; whereas immune reconstitution inflammatory syndrome, new onset anaemia, deaths and other severe or life-threatening events were labelled as serious.

*Mycobacterium tuberculosis* genotyping was performed with a spoligotyping commercial kit (Ocimum Biosolutions Ltd., Hyderabad, India) according to a previously described method, on relapse samples and corresponding baseline samples, to differentiate exogenous re-infection from endogenous reactivation [26].

### Endpoints

The primary endpoint was outcome at the end of initial treatment, as defined by the WHO: cure (smear- and culture-negativity at end of treatment in smear-positive pulmonary TB), treatment completed (no evidence of treatment failure or cure), treatment failure (smear- or culture-positivity in the last 2 months of treatment, or evidence of clinical and/or radiological worsening or partial response), or death [3]. The term ‘treatment success’ refers to the sum of ‘cure’ and ‘treatment completed’, and was used as the main comparative outcome, due to the inclusion of smear-negative and extrapulmonary cases in whom the ‘cure’ label cannot be applied.

Secondary endpoints were treatment outcome at the end of 24-month follow-up; duration of initial treatment; sputum smear- and culture-conversion rate in sputum-positive pulmonary TB patients; adverse events; and mortality in HIV-co-infected patients.

### Statistical analysis

Sample size calculation was as follows: an initial sample of 165 patients would be required in each group would be sufficient to detect a difference in treatment success of 15% (HIV negative = 85%, HIV positive = 70%, with two sided 5% significance level and 90% power), assuming 25% attrition. Primary analysis was by modified intention to treat, with on-treatment analysis for adverse events. All patients receiving at least one dose of ATT were included for treatment outcome analysis. Comparisons between groups were analysed using Fisher’s exact or Pearson chi-squared tests for categorical data, and unpaired t-test or Wilcoxon Rank-sum test for qualitative characteristics. Odds ratios were calculated for independent risk factors for mortality in the HIV-positive group. All analysis was carried out using Stata® version 11.

## Results

### Patients

**Figure 1:**
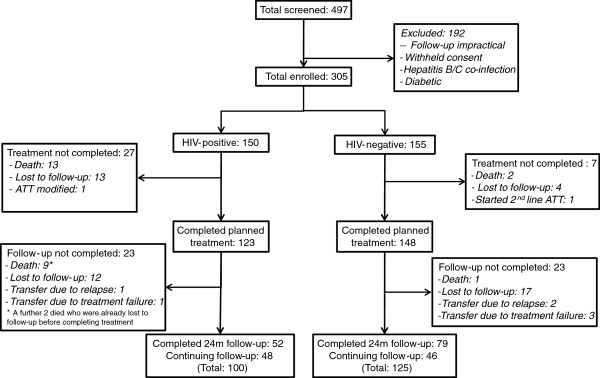
Study profile.

305 patients were enrolled from the 497 consecutively screened for eligibility; 155 were HIV-negative and 150 tested positive for HIV (Figure 1). End of treatment outcomes underwent modified intention to treat analysis: all patients undergoing any treatment were included. Follow-up based outcomes, also undergoing modified intention to treat analysis, included all except those still under regular follow-up (n = 211). In the context of adverse events, on treatment analysis was applied to all those receiving any ATT (n = 305).

Baseline characteristics for the two groups are compared in Table 1. Body mass index and serum bilirubin were not found to differ significantly. Foci of TB involvement in each group are shown in Table 2. Extrapulmonary involvement (p = 0 · 001) and dissemination (p < 0 · 001) were significantly more common in patients with HIV. For pulmonary TB patients, Tables 3 depicts the baseline sputum smear results and chest radiograph severity. The bacillary load was significantly higher in HIV-negative than HIV-positive smear-positive patients (P < 0 · 001).

**Table 1:** Baseline characteristics

**Characteristic**	**HIV + (n = 150)**	**HIV- (n = 155)**	**P-value**
Age	34.2 ± 8.3	27.2 ± 9.5	<0.001
Gender (M:F, %)	83:17	58:42	<0.001
BMI (kg/m^2^)	18.1 ± 2.8	17.9 ± 3.6	0.48
Haemoglobin (g/dL)	10.0 ± 2.2	11.1 ± 1.8	<0.001
Erythrocyte sedimentation rate (mm/hr)	49.4 ± 23.6	43.7 ± 21.4	0.03
Absolute lymphocyte count (/mm^3^)	2071 ± 1010	2435 ± 938	0.001
CD4 count (/mm^3^)	194 ± 167	593 ± 232	<0.001
Albumin (g/dL)	3.5 ± 0.7	4.2 ± 0.6	<0.001
Bilirubin (mg/dL)	0.65 ± 0.24	0.67 ± 0.16	0.48
Alanine aminotransferase (IU/L)	53.7 ± 42.5	39.2 ± 21.9	<0.001
Aspartate aminotransferase (IU/L)	48.2 ± 70.3	32.7 ± 21.0	<0.01
Uric acid (units)	5.7 ± 2.7	5.1 ± 1.5	<0.01
Viral load (log copies/mL)	5.23 (4.74-5.76)		

Mean +/− standard deviation except median (inter-quartile range) in viral load.

**Table 2:** Systemic foci of TB involvement

	**HIV + (n = 150)**	**HIV- (n = 155)**	**Overall (n = 305)**
**Pulmonary TB**	**89 (59.3%)**	**119 (76.8%)**	**208 (68.2%)**
*Pulmonary only*	*49 (32.7%)*	*111 (71.6%)*	*160 (52.5%)*
*Miliary*	*2 (1.3%)*	*2 (1.3%)*	*4 (1.3%)*
Disseminated*	*38 (25.3%)*	*6 (3.9%)*	*44 (14.4%)*
**Extrapulmonary TB**	**61 (40.7%)**	**36 (23.2%)**	**97 (31.8%)**
*Lymph node*	*35 (23.3%)*	*23 (14.8%)*	*58 (19.0%)*
*Pleural*	*5 (3.3%)*	*4 (2.6%)*	*9 (3.0%)*
*Intestinal*	*0*	*1 (0.6%)*	*1 (0.3%)*
*Genitourinary*	*0*	*1 (0.6%)*	*1 (0.3%)*
*Peritoneal (ascites)*	*0*	*1 (0.6%)*	*1 (0.3%)*
Disseminated*	*21 (14%)*	*6 (3.9%)*	*27 (8.9%)*

* Dissemination was defined as involvement of two or more non-contiguous organ sites [27]. Patients were labelled as ‘pulmonary, disseminated’ in case of presence of concomitant pulmonary and distant extrapulmonary disease, or ‘extrapulmonary, disseminated’ if there was evidence of dissemination without any lung parenchymal involvement.

**Table 3:** Baseline sputum smear and culture results for pulmonary TB patients

***n (%)***	**HIV + (n = 89)**	**HIV- (n = 119)**	**Overall (n = 208)**
**Smear results**			
Negative	24 (27.0%)	9 (7.6%)	33 (15.9%)
Unable to produce sputum	19 (21.3%)	7 (5.9%)	26 (12.5%)
Total positive	46 (51.6%)	103 (86.6%)	149 (71.6%)
*Scanty*	*8 (17.4%)*	*4 (3.9%)*	*12 (8.1%)*
*1+*	*22 (47.8%)*	*24 (23.3%)*	*46 (30.9%)*
*2+*	*6 (13.0%)*	*25 (24.3%)*	*31 (20.1%)*
*3+*	*8 (17.4%)*	*50 (48.5%)*	*58 (38.9%)*
*Grading unavailable (Positive)*	*2 (4.3%)*	*0*	*2 (1.3%)*
**Culture results**			
Negative	24 (27.0%)	5 (4.2%)	29 (13.9%)
Unavailable^+^	24 (27.0%)	10 (8.4%)	34 (16.3%)
Positive	41 (46.1%)*	104 (87.4%)*	145 (69.7%)
**Chest radiograph severity†**			
No infiltrates	10 (11.2)	2 (1.7)	12 (5.8)
Minimal lesions	25 (28.1)	27 (22.7)	52 (25.0)
Moderately advanced	38 (42.7)	61 (51.3)	99 (47.6)
Far advanced	16 (18.0)	29 (24.4)	45 (21.6)

* One HIV-negative patient was smear-negative, culture-positive; four HIV-positive patients were smear-positive but culture negative, and one HIV-positive individual was smear-positive, but their culture result was not available.

^+^ This includes those unable to produce sputum despite sputum induction (see 'smear results' section of Table 3), as well as those whose sputum culture results were not available for other reasons.

† According to the National Tuberculosis Association of the USA classification [22].

The basis of diagnosis for each group is shown in Table 4. Using the classification described above, 74 (49 · 3%) of the HIV-positive and 121 (78 · 1%) of HIV-negative diagnoses were ‘definitive’. Of the 83 (27 · 2%) ‘radio-clinical’ cases (diagnosed on the basis of radiological findings and clinical features), all but 11 of these displayed full response to treatment – of those who did not, six died, four were lost to follow-up during treatment, and one failed treatment. Extensive attempts were made to rule out other diagnoses.

**Table 4:** Basis of diagnosis of TB

**Basis of diagnosis *n (%)***	**HIV + (n = 150)**	**HIV- (n = 155)**	**Overall (n = 305)**
**Smear/culture**^**+**^	70 (46.7)	120 (77.4)	190 (62.3)
**PCR**^**+**^	4 (2.7)	1 (0.6)	5 (1.6)
**Cyto-/Histopathology**	11 (7.3)	15 (9.7)	26 (8.5)
**Chemistry (raised fluid ADA levels)**	0	1 (0.6)	1 (0.3)
**Radio-clinical**	65 (43.3)	18 (11.6)	83 (27.2)

^**+**^ Constitute *definitive* diagnosis.

N.B. in case of more than one diagnostic criterion being suggestive of TB, that with highest value was taken, in the order: smear/culture > PCR > cyto-/histopathology > chemistry > radio-clinical.

### End of treatment outcome

Treatment outcome at the end of ATT for each group is detailed in Table 5. Treatment success was achieved in 76 · 7% of the HIV-positive and 93 · 5% of the HIV-negative patients (p <0 · 001). There were nine HIV-positive treatment failures; four in the HIV-negative group (p = 0 · 16), of which six (two pulmonary) and four (three pulmonary) were microbiologically confirmed, respectively. One HIV-infected patient who failed initial ATT, was well after re-treatment with a 'category II' regimen, but later relapsed, and after the end of scheduled follow-up was diagnosed with multi-drug resistant (MDR) pulmonary TB. One HIV-negative patient failed the re-treatment regimen and was subsequently diagnosed as having MDR pulmonary TB.

**Table 5:** Treatment outcome at end of initial ATT

**TREATMENT OUTCOME *number (percent)***	**HIV + (n = 150)**	**HIV- (n = 155)**	**P-value**
***Treatment success***^***+***^	***115 (76.7)***	***145 (93.5)***	***<0.001***
**Treatment completed**	85 (56.7)	45 (29.0)	
**Cure**	30 (20.0)	100 (64.5)	
**Treatment failure**	**8 (5.3)**	**4 (2.6)**	**0.16**
**Default**	4 (2.7)	0	
**Lost to follow-up**	9 (6.0)	4 (2.6)	
**Death**	**13 (8.7)**	**2 (1.3)**	**<0.01**
**Treatment modified**	1 (0.7)	0	

+ Sum of *cure* and *treatment completed*.

Thirteen HIV-positive and two HIV-negative patients died while on treatment (p < 0 · 01). Thirteen (four defaulters, nine untraceable) HIV-positive patients were lost to follow-up; as were four HIV-negative patients (all untraceable). One patient with HIV had to have their treatment modified permanently due to isoniazid-induced psychosis.

A total of 35 (23 · 3%) HIV-positive patients required extensions of ATT, whereas only 13 (7 · 8%) of the HIV-negative patients had their ATT extended (p = 0 · 001). Each group had a median additional duration of 1 month.

Comparison of baseline BMI and laboratory parameters with those at 6 months of treatment is provided in Table 6. Serum albumin and CD4 counts increased significantly in HIV-positive patients.

**Table 6:** Changes in various parameters between baseline and 6 months of treatment

	**HIV+**		**HIV-**	
**Parameter**	**Pre**	**Post**	**Pre**	**Post**
BMI, mean, kg/m^2^	18.1	19.8	17.9	19.7
Haemoglobin, mean, g/dl	10.0	12.3	11.1	13.2
**ESR, mean, mm/h**	**49.4**	**34.1**	**43.7**	**24.2**
**Albumin, mean, g/dl**	**3.5**	**4.3**	**4.2**	**4.7**
**CD4 cell count, mean, cells/mm**^**3**^	**194.1**	**330.8**	**593.2**	**676.5**
HIV viral load, median, copies/ml	170339	400	-	-

**Bold type** = statistically significant difference in degree of change between the two groups (p < 0.05).

Subgroup analysis was performed on initial treatment outcomes of sputum smear-positive pulmonary TB patients with and without HIV co-infection, who had completed ATT (Table 7). One hundred of 105 patients (95 · 2%) in the HIV-negative group and 36 of 52 (69.2%) HIV-positive patients had treatment success (p < 0 · 001).

**Table 7:** Treatment outcome in smear-positive pulmonary TB patients at end of initial ATT

**TREATMENT OUTCOME *number (percent)***	**HIV + (n = 52)**	**HIV- (n = 105)**	**P-value**
***Treatment success***^**+**^	***36 (69.2)***	***100 (95.2)***	***<0.001***
**Treatment completed**^**^**^	6 (11.5)	5 (4.8)	
**Cure**	30 (57.7)	95 (90.5)	
**Treatment failure**	2 (3.8)	2 (1.9)	
**Lost to follow-up**	8 (15.4)	2 (1.9)	
**Death**	6 (11.5)	1 (1.0)	

^+^ Sum of *cure* and *treatment completed.*

^^^ These patients were either diagnosed by bronchoscopy and smear positive aspirate, or unable to produce sputum to provide evidence of sputum conversion and therefore cure, but had clinical and radiological resolution at end of treatment.

Sputum smear (32/38 [84.2%] HIV+, 64/80 [80.0%] HIV-, p = 0.58) and culture (29/34 [85.3%] HIV+, 71/88 [80.7%] HIV-, p = 0.55) conversion proportions in sputum-positive pulmonary patients at two months (up to 9 weeks) were found to be similar among the two groups.

### Follow-up

At the time of analysis, a total of 123/150 (82%) of the HIV-positive and 148/155 (95 · 5%) HIV-negative patients had completed planned treatment of 6 to 9 months (Figure 1).

Table 8 shows treatment outcome at 24 months follow-up (i.e. excluding patients under active follow-up, but including deaths and loss to follow-up). The mean follow-up time for HIV-positive and –negative patients was 14 · 1 ± 8 · 73 months and 17 · 8 ± 7 · 80 months, respectively. Attrition rates were 24.5% in the HIV-positive group, and 19.3% of HIV-negative patients, despite attempts (telephone, home visits) to find those who were lost to follow-up.

**Table 8:** Treatment outcome at 24 months follow-up*

**TREATMENT OUTCOME *number (percent)***	**HIV + (n = 102)**	**HIV- (n = 109)**	**P-value**
**Treatment success**^**+**^	**41 (40.2)**	**79 (72.5)**	**P < 0.001**
***Treatment completed***	*37 (36.3)*	*38 (34.9)*	
***Cure***	*4 (3.9)*	*41 (37.6)*	
**Treatment success [excluding those lost to follow up and defaulters]^**	**41 (53.2)**	**79 (89.8)**	**P < 0.001**
**Treatment failure**	8 (7.8)	4 (3.7)	
**Relapse**	**5 (4.9)**	**2 (1.8)**	
**Default**	4 (3.9)	0	
**Lost to follow-up**	21 (20.6)	21 (19.3)	
**Death**	**22 (21.6)**	**3 (2.8)**	**P < 0.001**
**Treatment modified**	1 (1.0)	0	

* Includes patients completing 24 months follow-up and those being labelled as death, lost to follow-up, failure and relapse – i.e. does not include patients still under active follow-up.

^**+**^ Sum of *cure* and *treatment completed.*

^ In this outcome, the 'default' and 'lost to follow up' groups have been removed from the cohort, so that for HIV + n = 77, and for HIV- n = 88.

Uni- and multivariate analysis of independent variables found no predictor of unfavourable outcome (i.e. any outcome other than treatment success) in the two groups. Variables included various blood test results at baseline (erythrocyte sedimentation rate, serum albumin, haemoglobin, CD4 cell count), chest X-ray severity, sex, age, presence of dissemination of TB, body mass index, and whether the TB was pulmonary or extrapulmonary.

The median time of relapse after treatment was six months. Relapse rate in percentage per person year observed (PYO) was calculated: in HIV-positive patients, 3 · 48% PYO (95% confidence interval [CI]: 0 · 43-6· 54%), and 0 · 91% PYO (CI: 0 · 35-2 · 18%) in HIV-negative patients [p = 0 · 09]. Of the five HIV-positive relapses, three were pulmonary; the two HIV-negative patients both suffered pulmonary relapses. Of the four HIV-positive pulmonary TB patients, 3 had different spoligotyping genotypes suggesting exogenous re-infection while one had similar genotype suggesting endogenous reactivation. One patient in the HIV-negative group had similar genotype.

### HIV-specific outcomes

Of the HIV-positive patients, all but one were started on highly active ART (one died before commencement). The median interval between starting the ATT and ART was 30 days (interquartile range: 17–60 days). Zidovudine-based therapy was given to 65%, the remaining 35% (53/150) received stavudine.

Mean rise in CD4 cell count and median fall in log plasma HIV viral load over the TB treatment phase were statistically significant (P < 0.001).

Co-trimoxazole prophylaxis was given in 134/150 (89 · 3%). Fourteen patients were found to have opportunistic infections other than TB, some with multiple infections. The majority of these were present at baseline assessment.

Uni- and multivariate analysis of independent risk factors for mortality in the HIV-positive group found only baseline CD4 cell count to be significant. Patients with CD4 cell count of less than 100 cells/mm^3^ had an odds ratio of 8 · 43 for mortality (p = 0 · 013; CI: 1 · 58-44 · 95).

### Adverse events

Table 9 details adverse events in the two groups while on treatment (totals and selected events). A similar number of non-serious adverse events were encountered in both groups (p = 0.851), while significantly more serious adverse events were observed in the HIV-positive patients (p < 0.001). Anaemia prompted switch from zidovudine to stavudine, while drug induced hepatotoxicity was treated by temporary ATT modification. Besides deaths and IRIS (see below), no other serious adverse event was observed.

**Table 9:** Adverse events in each group while on ATT

	**HIV + (n = 150)**	**HIV- (n = 155)**	**Overall (n = 305)**
Total patients experiencing adverse event(s)	69 (46.0%)	74 (47.7%)	143 (46.9%)
Total patients experiencing serious adverse event(s)	44 (29.3%)	3 (1.9%)	47 (15.4%)
IRIS*	14 (9.3%)	0	14 (4.6%)
Anaemia (new on treatment)*	9 (6.0%)	0	9 (3.0%)
Drug-induced hepatotoxicity*	5 (3.3%)	0	5 (1.6%)
Isoniazid-induced psychosis*	1 (0.7%)	0	1 (0.3%)
Stevens-Johnson syndrome*	1 (0.7%)	0	1 (0.3%)
Peripheral neuropathy*	2 (1.3%)	0	2 (0.7%)
Haemoptysis*	0	6 (3.9%)	0
Itch	9 (6.0%)	2 (1.3%)	11 (3.6%)
Rash	11 (7.3%)	2 (1.3%)	13 (4.3%)
Nausea/vomiting	47 (31.3%)	71 (45.8%)	118 (38.7%)
Diarrhoea	17 (11.3%)	24 (15.5%)	41 (13.4%)
Abdominal pain	17 (11.3%)	26 (16.8%)	43 (14.1%)

* Classified as serious adverse events.

Immune reconstitution inflammatory syndrome (IRIS) occurred in 14 patients at a median of 76.5 days after commencing ART; all were mild and did not require discontinuation of treatment. There was a significant increase in median CD4 cell count (p = 0 · 001) and decrease in mean log plasma viral load (p < 0 · 0001) between pre-IRIS and post-IRIS values.

In total, 22 HIV-positive and 3 HIV-negative patients died. Of the HIV-positive deaths, 5 were within the first 2 months, a further 8 on treatment, and 9 were during follow-up; the median time of death was 5.5 months after enrolment. Four deaths (three HIV-positive, one HIV-negative) were confirmed as due to TB. Insufficient details were available for the remaining patients to determine causality, as they died at home or at a different hospital.

## Discussion

The proportion of HIV-negative patients achieving treatment success was much higher than HIV-positive patients: at the end of treatment, 93.5% versus 76.7% (P < 0.001); at the end of treatment in those with sputum positive pulmonary TB, 95.2% versus 69.2% (P < 0.001); and at 24 months follow up excluding those lost to follow up, 89.8% versus 53.2% (P < 0.001). Multivariate analysis did not reveal any independent risk factor for not achieving treatment success within each group.

Higher treatment success in HIV-negative and higher mortality in HIV-positive patients in the present study are in agreement with previous studies [8,13]. Indeed, the end of treatment mortality rate in the present study’s co-infected patients (8 · 1%) is lower than most previous figures – Khan et al’s meta-analysis quotes 15% [16]. In many studies, no patients received ART (unlike universal highly active ART in the present one), which may explain higher mortality. Abdool Karim et al. found in 2010 that ART during ATT reduced mortality by 56%, to just 5.4% in median 12.1 months follow-up [2]. Interestingly, recent studies without concurrent ART quote 10 · 4% [12] and 10 · 9% [18], which suggests the fall in mortality may be due in part to other improvements in HIV/TB care.

Mortality was largely after the intensive phase of ATT, with half of the HIV positive patients dying in the follow-up stage, which has been suggested previously [2,11,12]. Only four deaths in the present study were attributable to TB. These observations may implicate HIV/AIDS as a more important factor for mortality than TB, though paucity of death causation data means this assumption is difficult to make. However, lower nadir CD4 counts in patients who subsequently died support this hypothesis, which is often related to delayed presentation, described previously at our Centre [20].

Multivariate analysis revealed those with CD4 count < 100 cells/mm^3^ had 8-fold higher risk of dying. This has been shown previously [2,12], and suggests that immunological effects of HIV need close attention in TB-HIV co-infection, with early ART commencement being advocated by most bodies [2,3]. It may also mean that this subgroup may be considered for targeted interventions such as daily therapy, extended duration or otherwise.

Treatment failure and relapse rates were not significantly different in HIV-positive and HIV-negative patients. Previous publications are divided on these with regards to HIV status [5]. Most studies have shown similar failure rates regardless of HIV status [13,27], which is observed in the present study. Some studies report relapse being more common in HIV co-infection [11,28]. Pooled data show a higher relapse rate (12% PYO) in HIV-positive individuals than reported in this study (3 · 48% PYO) [16]. Spoligotyping in HIV-positive patients revealed three out of four having exogenous reinfection with TB – reports of the proportion being reinfected vary, however, the number of relapses are too small in this case to make any conclusions. This may reflect inadequate airborne infection control measures.

Sputum smear and culture conversion rates at 2 months were found to be similar regardless of HIV status. The 2-month culture conversion rate for HIV-positive individuals (85 · 3%) was in accordance with earlier reports, with a range of 74-95% being quoted [11,17,18].

Extension of ATT of 1–3 months was required in 35 of the HIV-positive patients, but only 13 in the HIV-negative group (median 1 month). A trial comparing 6 with 9 months thrice weekly ATT demonstrated lower bacteriological recurrence with 9 months treatment, but found no other benefits [12]. Hence it is unclear whether longer treatment would be beneficial.

HIV-infected patients were started on ART a median of 30 days after ATT, with good immunological and virological response. This is representative of present care of such patients according to recent WHO guidance stating all people with HIV-TB co-infection should be started on ART early in the ATT course [3]. It correlates well with other studies looking at outcomes of HIV-TB co-infection with early/concurrent ART [2,29].

With regards to safety, a similar frequency of non-serious adverse events was found in each group; though this seems higher than in several studies, reporting up to 37% of patients experiencing any event [12,13,17]. Such events were all self-limiting or easily treated, and may reflect the close monitoring in this study. The significantly higher incidence of serious adverse events in the HIV-positive group was mostly made up of deaths and HIV-specific events. The 29.3% figure observed is similar to that observed in the 'integrated therapy' group of Abdool Karim et al's recent study [2]. In addition, findings of the present study are similar to previous reports of low hepatotoxicity figures with thrice weekly treatment regimens [30].

The study depicts an up-to-date comparison of outcomes of thrice weekly six-month ATT in HIV-positive and -negative individuals. Strengths include: supplementation of currently lacking follow-up data on outcomes of intermittent ATT in HIV co-infected patients, especially in terms of relapse rates and late mortality; the real-life situation has been studied, with inclusion of smear-negative and extrapulmonary patients, concurrent ART, and preservation of the national TB programme’s treatment protocol; and surveillance results, including sputum culture conversion rate and post-ATT follow-up, in Indian patients (currently absent from national programme reports).

Limitations of this study include: 1) Lack of routine DST in order to screen for drug resistance: this is not universally recommended for patients with a new TB diagnosis by Indian or WHO guidelines [3,24], but is increasingly being used in research settings [5,12]. 2) The high attrition rate, especially in the HIV-positive group and after initial treatment, may have impacted on completeness of follow-up data. This is a common problem in resource-limited settings, as highlighted previously at this institute’s ART clinic [20]. 3) A significant proportion of patients were not definitive (bacteriologically confirmed) cases of TB, despite thorough investigation to exclude other diagnoses and most having response to treatment. 4) The study was observational, with resulting lack of control of confounding factors, evidenced by baseline heterogeneity. 5) As numbers of relapse and failure cases were small, it is difficult to judge differences between the two groups with confidence in this respect.

## Conclusions

Intermittent ATT is desirable in developing country settings to maintain direct observation with minimal strain on stretched health systems. However it has been poorly studied [31]. The present study shows excellent outcomes and safety of thrice-weekly ATT in HIV-negative individuals, confirming its adequacy in these patients [3], though acquired rifampicin resistance may be promoted, which needs detailed study [3,5,12]. Outcomes in HIV co-infection do not seem to be as good, with lower treatment success and higher mortality being observed, though with adequate safety. This should prompt further focussed study on treatment options for these patients, such as a trial starting at this institute examining outcomes of daily versus thrice-weekly therapy. National programme coordinators should re-visit guidelines, and investigate whether therapy changes in HIV co-infection should be universal, as recommended by the WHO [3], or in specific subgroups, such as those with low CD4 counts, to balance attempts to reduce morbidity and mortality in TB-HIV co-infection with awareness of resource constraints.

## Abbreviations

ART: Anti-retroviral therapy; ATT: Anti-tuberculosis therapy; CI: Confidence interval; DOTS: Directly observed treatment, short course; DST: Drug susceptibility testing; ELISA: Enzyme-linked immunosorbent assay; HIV: Human immunodeficiency virus; IRIS: Immune reconstitution inflammatory syndrome; MDR: Multi-drug resistant; PCR: Polymerase chain reaction; PYO: Person year(s) observed; TB: Tuberculosis; WHO: World Health Organization.

## Competing interests

All authors: no competing interests.

## Authors' contributions

SKS designed the study. SKS, KM, SD, SR, BS, RV, DG and SS collected data. SKS, KM, BS and VS analysed the data and contributed to the first draft of the manuscript. All authors finalised, read and approved the final manuscript.

## Pre-publication history

The pre-publication history for this paper can be accessed here:

http://www.biomedcentral.com/1471-2334/13/468/prepub
